# Key Early Changes in Oral Squamous Cell Carcinogenesis Are Accelerated by Ectopic BMI1 Expression

**DOI:** 10.1158/2767-9764.CRC-25-0580

**Published:** 2026-01-20

**Authors:** Jorge Baquero, Xiao-Han Tang, Daniel Galke, Theresa Scognamiglio, Tuo Zhang, Lorraine J. Gudas

**Affiliations:** 1 Department of Pharmacology, Weill Cornell Medical College, New York, New York.; 2 Meyer Cancer Center, Weill Cornell Medicine, New York, New York.; 3 Department of Pharmacology, Weill Cornell Graduate School of Biomedical Sciences, New York, New York.; 4 Department of Pathology, Weill Cornell Medical College, New York, New York.; 5 Weill Cornell Genomics Core Facility, Weill Cornell Medical College, New York, New York.

## Abstract

Although 5-year relative survival rates for oral squamous cell carcinoma (OSCC) have moderately increased in the last 30 years, most patients are diagnosed during the later stages of the disease. B cell–specific Moloney murine leukemia virus integration site 1 (BMI1) is a biomarker of OSCC that is increased in epithelial basal stem cells (SC) of premalignant oral lesions. However, the molecular functions of BMI1 in early-stage OSCC have not been fully elucidated. In this study, we used a transgenic mouse line (KrTB) that overexpresses BMI1 in the tongue epithelial SCs to delineate BMI1 actions during these early stages. We observed more oncogenic changes in mice with ectopic BMI1 expression after only a short, 4-week treatment with the carcinogen 4-nitroquinoline 1-oxide (4-NQO). For example, we detected increased proliferation, oxidative stress, and expression of multiple transcripts and proteins linked to human OSCCs in murine tongue epithelia with high, ectopic BMI1 expression. Furthermore, increases in mRNAs encoding multiple metabolic targets, such as SLC16A3, PKM, and GPI1, were greater upon BMI1 overexpression with 4 weeks of 4-NQO treatment. In a human OSCC model (SCC-25 cell line) in which we deleted the BMI1 gene, we observed decreases in proliferation, oxidative stress, and expression of the glycolysis-associated protein GLUT1. Thus, BMI1 expression leads to increases in key features of early-stage, carcinogen-induced tumorigenesis, including metabolic reprogramming. Consequently, limiting BMI1 could be a potential target for cancer prevention approaches that merits further consideration and additional functional studies.

**Significance:**

Most OSCC diagnoses occur in advanced stages. Our data indicate that BMI1 overexpression, as detected in premalignant oral lesions, increases proliferation, oxidative stress, and expression of human OSCC-associated targets in our murine model after a short, 4-week carcinogen treatment. Thus, BMI1 could be a potential target for cancer prevention approaches.

## Introduction

Head and neck cancers, which include cancers of the oral cavity, pharynx, and esophagus, remain the sixth most prevalent cancer type in the world in terms of incidence (1). In the United States alone, about 59,660 new cases of head and neck cancers and almost 13,000 related deaths are estimated in 2025 (2). Squamous cell carcinoma is the most common cancer in the head and neck mucosa (3, 4). More than 90% of all oral cavity cancers originate from squamous tissues and hence are known as oral squamous cell carcinomas (OSCC; refs. 5–7). Five-year relative survival rates for all oral cavity cancers have moderately increased from 58% to 69% in the last 30 years (2). Despite these advances, OSCCs still have high recurrence rates and often become resistant to conventional therapies (7–9). Furthermore, most patients are diagnosed during the later stages of the disease (5, 10, 11). Thus, identification of early biomarkers and new therapeutic targets is vital and requires a wider understanding of the molecular factors involved in OSCC onset and progression (10, 11).

Stem cells (SC) are crucial contributors to development and homeostasis of oral cavity epithelia (12), as they divide asymmetrically to give rise to all progeny cells that maintain this layer (13). We previously reported that labeled basal SCs are present in invasive squamous cell carcinomas and are the cells of origin of tongue tumors (14). These cancer SCs (CSC) are critical for tumor initiation and growth, metastasis, and recurrence (4, 15). CSCs have the ability to self-renew and to differentiate into other cell types; they can reside within specific tumor microenvironments that protect them from chemotherapy and radiotherapy (7). Accordingly, developing therapies that target CSCs is a promising approach for OSCC treatment (4, 7, 16).

B cell–specific Moloney murine leukemia virus integration site 1 (BMI1) is a key nuclear protein overexpressed in OSCCs (8, 17, 18). BMI1 is a major component of the mammalian polycomb repressive complex 1 (PRC1), a multi-protein complex involved in the monoubiquitination of histone H2A to repress the expression of downstream targets (19–21). BMI1 is expressed in almost all tongue basal SCs (22); thus, it is involved in the epigenetic regulation of SC functions, including proliferation, development, and differentiation (19–21). Moreover, BMI1-positive CSCs are slow-cycling, tumor initiating SCs (4) which can give rise to entire regions in OSCCs (17) and are responsible for mediating lymph node metastasis, therapy resistance, and tumor relapse (16). Analyses in human samples show that increased BMI1 expression is detected early during oral carcinogenesis, including in dysplastic oral leukoplakia (18, 23).

We previously developed a transgenic mouse line [K14-rtTA;TRE-FLBmi-1 (KrTB)] in which ectopic BMI1 is overexpressed in tongue basal epithelial SCs upon doxycycline (DOX) treatment (24). We reported that ectopic BMI1 expression aggravates OSCCs by increasing the percentage of invasive lesions (25). Furthermore, BMI1 overexpression promotes glycolysis during late oral tumorigenesis, when tumors are already visible on the tongues (25). We hypothesize that increased BMI1 expression, as detected in dysplastic lesions, could accelerate key changes observed during OSCC. Thus, in this study we use this KrTB line in a 4-nitroquinoline 1-oxide (4-NQO)-induced carcinogenesis model (25) to identify these early molecular, carcinogenic changes upon BMI1 overexpression. We complement these studies with the use of a human OSCC cell line SCC-25 in which we deleted the BMI1 gene.

## Materials and Methods

### KrTB and Kr mice and doxycycline treatment

**Figure 1.**
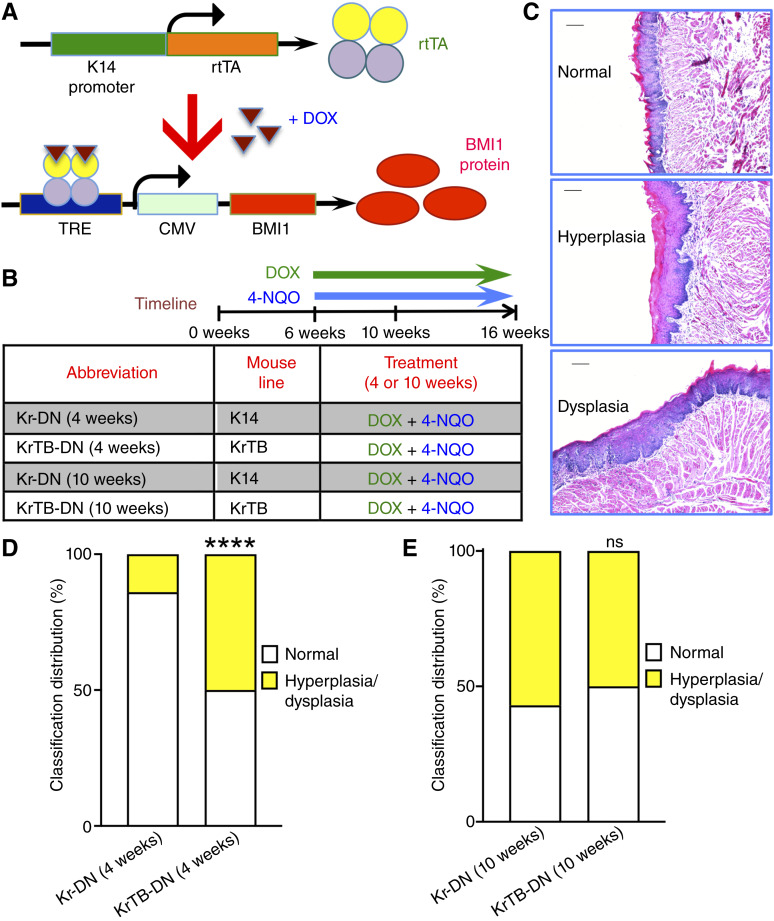
Tumor pathologic classification after 4-NQO treatment for 4 weeks reveals more oncogenic changes in mice with ectopic BMI1 expression. **A,** Schematic of the DOX-inducible expression system (KrTB) used to overexpress ectopic BMI1 in murine tongue epithelial basal stem cells. **B,** Timeline outlining the different treatment groups for this experiment. Samples were collected at 10 or 16 weeks (4- or 10-week treatments). Samples from bolded groups [KrTB-DN (4w) and KrTB-DN (10w)] were also submitted for RNA-seq. **C,** Representative images of H&E-stained sections for normal tongue epithelium, hyperplasia, and dysplasia (100× magnification; scale bar, 100 μm). **D,** Graph of the distribution (%) of the most severe 4-NQO–induced lesion observed in each mouse by pathologic classification (at 4 weeks), with Kr-DN (4w) (*N* = 7) and KrTB-DN (4w) (*N* = 10) mice. **E,** Graph of the distribution (%) of the most severe 4-NQO–induced lesion observed in each mouse by pathologic classification (at 10 weeks), with Kr-DN (10w) (*N* = 7) and KrTB-DN (10w) (*N* = 10) mice. For **D** and **E**, statistical significance was determined with the *χ*^2^ test. ****, *P* < 0.0001.

KrTB mice (RRID: MGI_7840083, Fig. 1A) were described and characterized before (24–26). K14-rtTA mice (abbreviated as Kr, RRID: IMSR_JAX:008099) were obtained from The Jackson Laboratory. Six-week-old female Kr and KrTB mice were treated with 2 mg/mL DOX hyclate (Sigma-Aldrich; D9891-25G) added to the drinking water (24, 25) and replenished twice a week. DOX treatment continued for 4 or 10 weeks, after which the mice were killed. Before all mice were killed, DOX-containing water bottles were removed from cages for 1.5 hours and placed back for an additional 1.5 hours. The mouse lines, treatments, and abbreviations used are indicated (Fig. 1B). All tongues were dissected immediately after cervical dislocation.

### 4-NQO administration

Cohorts of female Kr and KrTB mice treated with DOX for 4 or 10 weeks (w) were simultaneously treated with 50 μg/mL 4-NQO (Sigma-Aldrich; D9891) in the drinking water [Kr-DN (4w), KrTB-DN (4w), Kr-DN (10w), and KrTB-DN (10w) groups, *N* = 7 to 10 per group, Fig. 1B], as previously described (14, 24, 25, 27–29). Mouse tongues were isolated 4 or 10 weeks after the initiation of DOX+4-NQO (DN) treatments.

### Histologic analysis of tongue lesions

For pathologic classification, a portion of tongue tissue was fixed in 4% paraformaldehyde (PFA; Sigma-Aldrich; #P6148) overnight. Tissues were processed and sectioned at 5 to 7 μm in the Weill Cornell Electron Microscopy and Histology Core. The histologic diagnosis of squamous neoplasia was performed by a board-certified head and neck pathologist (T.S.) on hematoxylin and eosin (H&E)-stained tissue samples in a blinded manner (14, 24, 25, 27–29). The most severe lesion in each mouse was categorized as hyperplasia or dysplasia (24, 25, 28, 29).

### RNA isolation, genome-wide RNA sequencing, and data analysis

Total RNA was isolated from ∼3 mg of tongue epithelia collected from four female mice/group [KrTB-DN (4w), KrTB-DN (10w), and KrTB-N (25w) groups, Supplementary Fig. S1A]. Epithelia were separated from the remainder of tongue tissues, saved in RNALater, and homogenized as described (25, 26). The RNeasy Micro Kit (Qiagen; 74004) was used for RNA isolation, following the manufacturer’s protocol. Final products were eluted from columns using 14 μL of UltraPure distilled water (25, 26). RNA integrity number measurements, cDNA library preparation, and deep sequencing were carried out by the Genomics Resources Core Facility of Weill Cornell Medical College (WCMC), as described (30). Differential expression analyses were performed using DESeq2 v1.6.3 (RRID: SCR_000154) as before (25, 26, 30). For these analyses, we compared the KrTB-DN (4w;) (Supplementary Table S1) and KrTB-DN (10w) groups (Supplementary Table S2) with a later time point group, KrTB-N (25w), which was processed and sequenced simultaneously and was included in a previous study (accession number GSE285101; ref. 25). The KrTB-N (25w) group only expressed *endogenous* BMI1 because it was not DOX-treated, and this group was treated with 4-NQO and allowed to develop lesions for a longer period of time (25 weeks), as described before (25). We also compared the KrTB-DN (10w) with the KrTB-DN (4w) group (Supplementary Fig. S1B). The RNA sequencing (RNA-seq) data for this study have been deposited in Gene Expression Omnibus (GEO; NCBI) repository (accession number GSE307391) and are publicly accessible.

### Immunohistochemistry, immunofluorescence, and immunohistochemistry/immunofluorescence quantification

Tissues for immunohistochemistry (IHC) and immunofluorescence (IF) were harvested immediately after cervical dislocation, fixed in 4% PFA overnight, and subsequently processed and sectioned. We performed IHC staining as described (31, 32). For IF staining, we performed deparaffinization, rehydration, antigen retrieval on paraffin-embedded sections, blocking, primary/secondary antibody incubations, Sudan Black B quenching, and mounting as previously described (29). The antibodies and conditions used in IHC and IF are shown (Supplementary Table S3). All images were acquired on a Nikon Eclipse TE2000-E microscope using NIS-Elements AR software. 200× magnification was used for all IHC and IF images. To quantify the intensity of staining, the signals for IHC and IF were analyzed using the Fiji (ImageJ) software [National Institutes of Health (NIH), RRID: SCR_003070], and values were normalized as indicated in figures and as previously described (29). All IHC quantifications were performed using four representative fields per section (as shown in each figure), with slides from *N* = 3 mice per experimental condition analyzed. For IF, we stained slides from three mice/group [Kr-DN (4w) and KrTB-DN (4w) groups only] and used four representative fields/mouse for quantifications. We report ratios of integrated density of HIF1α or GLUT1 (measured by Fiji) over integrated density of Hoechst signal in the same area, as previously described (29). Representative IF images are shown.

### cDNA generation and qRT-PCR analysis

Total RNA from Kr-DN (4w), KrTB-DN (4w), Kr-DN (10w), and KrTB-DN (10w) tongue epithelia was isolated as summarized above. Reverse transcription was performed using 0.5 μg of total RNA and qScript cDNA Synthesis Kit (Quanta Biosciences, #95047-100). qRT-PCR reactions (15 μL) were carried out using PerfeCTa SYBR Green SuperMix (Quanta Biosciences, #95053-02K). 1 μL of 1:10 diluted cDNA was used as template in all qRT-PCR reactions, except for reactions with HIF1A (2 μL) and SLC2A1 (3 μL) primers. Supplementary Table S4 includes the specific primer sequences for each gene used in qRT-PCR reactions. Primers were designed from the GRCm39/mm39 mouse genome assembly using the NCBI primer tool (ncbi.nlm.nih.gov/tools/primer-blast) and verified using the UCSC Genome Browser (genome.ucsc.edu). We designed our primers to amplify sequences spanning exon–exon junctions in which the primers are separated by at least one intron on the corresponding genomic DNA.

### Cell culture and two-step chromatin immunoprecipitation assay

The human OSCC line SCC-25 was obtained (at passage 38) and cultured in Dulbecco’s Modified Eagle Medium/Nutrient Mixture F-12 (DMEM/F-12; Thermo Fisher Scientific; 1132003) as before (33). No testing was performed for *Mycoplasma*. Chromatin immunoprecipitation (ChIP) was performed as before (26, 33, 34), using parental SCC-25 cells seeded at a density of 3 × 10^6^ cells/15-cm culture dish, and left to incubate for 2 days. To cross-link, cells were washed once with 1X PBS and then incubated with 10 mL of 1X PBS containing 2 μmol/L dissuccinimidyl glutarate (DSG; Thermo Fisher Scientific; 20593) and protease inhibitor (Sigma-Aldrich cOmplete Mini; 11836153001) and rocked for 45 minutes at room temperature. The DSG solution was then removed, and the plate was washed once more with 1X PBS before 10 mL of 1% formaldehyde in 1X PBS (+protease inhibitor) was added to the plate and rocked at room temperature for another 20 minutes. This cross-linking process was quenched by the addition of 1.6 mL of 1.25 mol/L glycine in dH_2_O and rocked at room temperature for an additional 5 minutes. The cells were scraped from the plate in 10 mL of 1X PBS and centrifuged at 1,500 rpm for 10 minutes at 4°C. The supernatant was decanted, and the resulting cell pellet was resuspended in 500 μL radioimmunoprecipitation assay buffer (RIPA; Thermo Fisher Scientific; 89900) supplemented with 10 μL protease inhibitor (Thermo Fisher Scientific; 78442) and 5 μL dithiothreitol solution (Thermo Fisher Scientific; 707265ML) per 1 mL RIPA buffer. The samples were then incubated on ice for 1 hour. The soluble chromatin was sonicated 10 times for 20 seconds each (Branson 150 Sonifier, setting 3), briefly centrifuging each sample in between sonications. The samples were then centrifuged at 13,200 rpm for 10 minutes at 4°C, and the resulting supernatants were collected into new tubes and labeled as soluble chromatin. The size of the DNA was confirmed by taking 25 μL of soluble chromatin and incubating at 65°C with 75 μL of ChIP elution buffer (50 μL Tris HCl pH 8, 100 μL 10% SDS, 2 μL 0.5 mol/L EDTA, and 848 μL dH_2_O) and 4 μL NaCl. The DNA was purified using Qiagen PCR Purification Kit (Qiagen; 28106) according to the manufacturer’s protocol. A 2.5% agarose gel was prepared, and the delinked DNA was loaded to verify the sample fragments were between 200 and 700 bps.

To prepare bead–antibody conjugates, M-270 Epoxy Dynabeads (Dynabeads Antibody Coupling Kit; Invitrogen; 14311D) were incubated at 37°C overnight with 10 μL per IP of rabbit monoclonal anti-Bmi1 antibody (Cell Signaling Technology; 6964S; lot 3; concentration: 87 μg/mL, RRID: AB_10828713) or 2.5 μL per IP of normal rabbit IgG (Santa Cruz; sc-2027; lot A3014; concentration: 400 μg/mL, RRID: AB_737197), following the manufacturer’s protocol. A volume of soluble chromatin equal to 10 μg of DNA was added to 50 μL of the bead–antibody conjugates along with 5.5 μL protease inhibitor, and the total volume was brought to 500 μL with IP buffer (0.01% SDS, 1.1% Triton-x100, 1.2 mmol/L EDTA, 16.7 mmol/L Tris-HCl pH 8.0, and 167 mmol/L NaCl). The samples were rotated overnight at 4°C. The next day, beads were sequentially washed with 700 μL of low-salt buffer (0.1% SDS, 1% Triton-x100, 2 mmol/L EDTA, 20 mmol/L Tris-HCl pH 8.0, and 150 mmol/L NaCl), high-salt buffer (same composition as low-salt buffer but with 500 mmol/L NaCl instead), LiCl wash buffer (0.25 mol/L LiCl, 1% NP-40, 1% deoxycholate, 1 mmol/L EDTA, and 20 mmol/L Tris-HCl pH 8.0), and twice with TE buffer (10 mmol/L Tris-HCl pH 8.0 and 1 mmol/L EDTA). After the last wash, 75 μL of TES buffer (10 mmol/L Tris-HCl pH 8.0, 1 mmol/L EDTA, and 0.25% SDS) and 3 μL of proteinase K (Qiagen; 1014023) were added to the beads. DNA fragments were eluted from immunocomplexes and beads by incubating at 65°C for 6 hours. The supernatants containing the DNA were separated into new tubes. DNA was then purified using a PCR purification kit (Qiagen; 28106) as before. These DNA samples were assayed by qPCR with primers designed from the GRCm39/mm39 mouse genome assembly (Supplementary Table S5) and normalized relative to diluted input DNA (1:50), as described before (26).

### Generation of BMI1 knockout SCC-25 cells (B#5 and B#6)

To obtain human OSCC cell lines with BMI1 knockout (KO), we used the Synthego online tool (Synthego) to design guide RNA (gRNA) sequences. We selected three gRNA sequences with the highest probability of BMI1 gene disruption, targeting exon 6 or 9. We seeded SCC-25 cells in 24-well tissue culture plates at a density of 50,000 cells/well, and after 24 hours, we treated cells with the three gRNA sequences (Supplementary Table S6), following the manufacturers’ instructions (Synthego). Briefly, we reconstituted each gRNA sequence with TE buffer (10 mmol/L Tris-HCl and 1 mmol/L EDTA, pH 8) to a 100 μmol/L stock concentration and prepared 30 μmol/L working solutions using the nuclease-free water provided. For each well, we prepared a mix containing 1.5 μL of one of the gRNA working solutions, 1.5 μL of the mRNA Cas9 nuclease (L-7206-100; TriLink Biotechnologies), 6 μL of Lipofectamine Messenger Max (LMRNA001; Invitrogen), and 200 μL of Optimem 1X (31985-070; Gibco). After mixing and incubating at room temperature for 5 minutes, we combined this solution with 800 μL DMEM, for a total of 1 mL, and dispensed it in the wells. One set of cells was incubated with a mix that did not contain any gRNAs, to serve as control (labeled “Par” for Parental). We transfected the SCC-25 cells for 9 ½ hours, then replaced the mix with 1 mL of DMEM/F12 media.

Transfected cells were allowed to recover for 2 days. This was followed by trypsinization and plating in six-well plates to allow cells to proliferate until >70% confluency. Then we extracted genomic DNA for testing and expanded the remaining cells. We performed Sanger sequencing on fragments amplified via PCR (primers used are shown in Supplementary Table S7) from genomic DNA of all treated cells. To assess genome editing, we compared fragments from gRNA-treated to control “Par” cells (used as reference genome). We used the online tool provided by Synthego, Inference of CRISPR Edits (ICE), which revealed potential editing in cells treated with one of the three gRNAs by providing a score indicative of the percentage of editing. The highest score resulting from this analysis was 38% for one of the gRNAs (gRNA B). We then performed single-cell selection from this polyclonal population by plating 200 cells per 10-cm dish and collected cells from 10 colonies after 2 weeks. We verified the success of CRISPR editing in each colony by Sanger sequencing followed by the Synthego ICE tool, as explained above. These analyses revealed two distinct lines, B#5 and B#6, with 97% and 82% editing efficiency and with 77% and 81% KO scores (proportion of cells with frameshift-inducing indels), respectively.

### Western Blotting using SCC-25 cells

SCC-25 samples were lysed in protein extraction buffer (0.125 mol/L Tris-HCl, pH 6.8, 2% SDS, and 2.5% beta-mercaptoethanol), and denatured by boiling. Total protein lysates (30 μg) were resolved by SDS-PAGE and transferred to a nitrocellulose membrane (cat. #162-0115; Bio-Rad). All cell lines (parental, B#5, and B#6) were run in triplicates and repeated on three independent occasions. Antibodies used are shown in Supplementary Table S8. Membranes were developed with enhanced chemiluminescence (cat. #32106; Thermo Fisher Scientific) for loading controls. Membranes were developed with SuperSignal West Pico PLUS chemiluminescence substrate (cat. #34580; Thermo Fisher Scientific) for target proteins. Signals were subjected to densitometry using ImageJ software for quantification.

### Proliferation in SCC-25 cells

Parental, B#5, and B#6 SCC-25 cells were plated on 24-well plates in triplicates at a density of 10,000 cells per well. Cells were counted using a Z1-D Beckman Coulter Counter 24 hours after seeding (T = 0 days) and then 1, 3, and 6 days after T0. Quantifications were performed from *n* = 6 replicates per experimental condition analyzed (experiments were repeated on two independent occasions).

### Detection of reactive oxygen species in SCC-25 cells

A general oxidative stress indicator (CM-H_2_DCFDA; C6827, lot #2539079) was purchased from Invitrogen and used in accordance with the manufacturer’s protocol, as before (26). Parental, B#5, and B#6 SCC-25 cells were plated at a density of 1 × 10^4^ cells/well in a 96-well glass bottom microplate (Cellvis, P96-0-N) and left overnight at 37°C. The next day, the ROS indicator (CM-H_2_DCFDA) was reconstituted to a 5 mmol/L stock solution in DMSO and diluted to a final working concentration of 5 μmol/L in 1X PBS. Cells were incubated with 100 μL of 5 μmol/L CM-H_2_DCFDA solution for 25 minutes at 37°C (loading time). Sets of parental, B#5, and B#6 cells were not incubated with the reactive oxygen species (ROS) indicator (for negative controls). After removing the ROS indicator, all cells were incubated with 100 μL of DMEM/F-12 medium for 20 minutes at 37°C (recovery time). NucBlue (Hoechst 33342; cat. #R37605, Invitrogen) was included in the media to label nuclei (2 drops/10 mL of media). Additional sets of parental, B#5, and B#6 cells initially incubated with the ROS indicator were also treated with H_2_O_2_ (100 μmol/L final concentration in DMEM/F-12) to stimulate oxidative activity (positive controls), as suggested by the manufacturer. After recovery time, cells were imaged using a Nikon Eclipse TE2000-E microscope and processed using the NIS-Elements AR software. 200× magnification was used for all cell images under the nine experimental conditions. Images were analyzed using the Fiji (ImageJ) software (NIH). Integrated density values for GFP signals (ROS detected) were calculated, as previously described (29), and normalized to the total number of nuclei per field (negative controls were not included in quantifications). Quantifications were performed using four representative fields per well, from *n* = 3 replicates per experimental condition analyzed, and with experiments repeated on two independent occasions.

### Statistics

Statistical analysis was performed on at least three samples from each group using GraphPad Prism 8 (RRID: SCR_002798). Data are represented as the mean ± SD and normalized as indicated in the figure legends. Classification distribution analyses (most severe lesion/mouse) include 7 to 10 independent biological replicates/group. RNA-seq analyses and ChIP experiments include four independent biological replicates/group. IHC, IF, and Western blotting (WB) experiments include three independent biological replicates/group. qRT-PCR experiments include at least three independent biological replicates/group. Count numbers for proliferation in SCC-25 cells include six replicates/experimental group. ROS detection experiments in SCC-25 cells include four representative fields/replicate and three replicates/experimental group. *P* values for IHC and ROS detection experiments were determined using one-way ANOVA followed by Tukey test. *P* values for IF, WB, qRT-PCR, and amounts of DNA IP’ed (ChIP on SCC25 cells) were determined using Welch *t* test. Significance of the distribution lesion pathology among the groups was analyzed using *χ*^2^ test. *P* values for proliferation in SCC-25 cells were determined using two-way RM ANOVA followed by Tukey multiple comparisons test. A *P* value of <0.05 was considered statistically significant. *P* values are indicated as *, 0.01 < *P* < 0.05; **, 0.001 < *P* < 0.01; ***, 0.0001 < *P* < 0.001; and ****, *P* < 0.0001.

### Ethical statement

The care and use of animals in this study were approved by the Institutional Animal Care and Use Committee of the WCMC and conformed to the US NIH guidelines for the humane care and use of laboratory animals.

## Results

### Tumor pathologic classification after 4-NQO treatment for 4 weeks reveals more oncogenic changes in mice with ectopic BMI1 expression

To elucidate the molecular changes that occur at early time points during OSCC carcinogenesis, we used our previously developed KrTB transgenic mouse line in which ectopic, FLAG-tagged BMI1 is overexpressed in the SCs of tongue epithelia (24–26). In KrTB mice, the expression of the reverse tetracycline-controlled transactivator (rtTA) is regulated by a truncated human keratin (K14) promoter (Fig. 1A). rtTA binds to the tetracycline response element (TRE) controlling the expression of FLAG-BMI1 only in the presence of DOX, which we add to the drinking water (24–26). We used the KrTB transgenic line in our immunocompetent mouse model of OSCC, based on the administration of the carcinogen 4-NQO in the drinking water to induce oral cancer in mice (28, 29, 35). In our experimental design, we also included Kr mice, which only express *endogenous* BMI1 even after DOX addition and 4-NQO treatment. These experimental groups, treated with 4-NQO for either 4 or 10 weeks, are defined in Fig. 1B.

To assess how BMI1 overexpression alters OSCC development, we evaluated H&E sections from tongue samples of all four experimental groups (Fig. 1B). The most severe lesions observed in each mouse after 4- or 10-week 4-NQO treatments were classified as hyperplasia or dysplasia by a board-certified pathologist (T.S.), and representative images are shown (Fig. 1C; refs. 24–26). After 4 weeks of 4-NQO, we found higher percentages of mice with hyperplasia/dysplasia in the KrTB-DN (4w) group (50.0%, *P* < 0.0001) compared with the Kr-DN (4w) group (14.3%; Fig. 1D). However, after 10 weeks of 4-NQO, the percentages of mice with hyperplasia/dysplasia in the KrTB-DN (10w) group matched those reported in the Kr-DN (10w) group (Fig. 1E). These results show that BMI1 overexpression accelerates early OSCC-related pathologic changes in tongue epithelia after carcinogen treatment.

### Increases in proliferation and oxidative stress occur very early after 4-NQO addition upon BMI1 overexpression

To gain insight into the early biological changes in OSCC that are accelerated by BMI1 overexpression, we performed genome-wide RNA-seq analyses on samples from KrTB-DN (10w) versus KrTB-DN (4w) tongue epithelia. We also compared each of these two groups with KrTB-N (25w) (accession number GSE285101), as changes associated with 4-NQO–induced tumorigenesis are fully detectable at 25 weeks in the presence of *endogenous* BMI1 (25). Indeed, the KrTB-N (25w) group only expressed *endogenous* BMI1 (i.e., No DOX) and was treated with 4-NQO and allowed to develop visible tongue lesions for a longer period of time (25 weeks, 15 weeks after 10 weeks of 4-NQO treatment; ref. 25). Thus, with these comparisons, we aimed to recognize the accelerated changes upon ectopic BMI1 overexpression. All experimental groups used for RNA-seq analyses are defined in Supplementary Fig. S1A. With these comparisons, KrTB-DN (4w) versus KrTB-N (25w) for Supplementary Table S1 and KrTB-DN (10w) versus KrTB-N (25w) for Supplementary Table S2, we identified the mRNA targets that are further increased/decreased early in OSCC upon BMI1 overexpression.

Our RNA-seq data revealed multiple transcripts associated with human OSCC (36) that are increased in KrTB-DN (10w) compared with KrTB-DN (4w) tongue epithelia (Supplementary Fig. S1B). These genes include S100A9, S100A8, LCN2, IL23A, and KLK6 (Supplementary Fig. S1B), all linked to human OSCC initiation and/or progression (37–40). Thus, during 4-NQO–induced tumorigenesis, the increased presence of hyperplasia/dysplasia observed at 4 weeks when BMI1 is overexpressed is followed by increased expression of OSCC-associated genes at 10 weeks as well.

Next, increases in transcripts involved in cell-cycle regulation and DNA replication (key drivers of cell proliferation), as well as changes in transcripts required in the response to oxidative stress, occur early during tumorigenesis in our 4-NQO model (36). In this study, our RNA-seq analyses indicated that several transcripts involved in cell proliferation were further increased in the KrTB-DN (4w) and KrTB-DN (10w) groups compared with the KrTB-N (25w) group (Supplementary Fig. S2). Likewise, changes in various transcripts involved in ROS regulation, such as decreased peroxiredoxin 5 (PRDX5) levels, took place in the KrTB-DN (4w) and KrTB-DN (10w) groups compared with the KrTB-N (25w) group (Supplementary Fig. S3).

**Figure 2.**
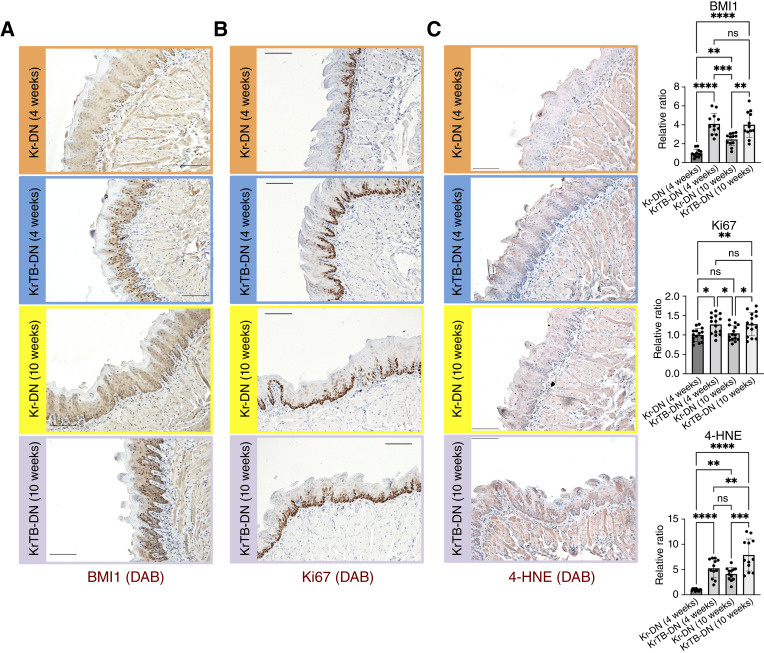
Increases in proliferation and oxidative stress occur very early after 4-NQO addition upon BMI1 overexpression. **A,** BMI1, (**B**) Ki67, and (**C**) 4-HNE IHC stainings in Kr-DN (4w), KrTB-DN (4w), Kr-DN (10w), and KrTB-DN (10w) tongue epithelia (200×; scale bar, 100 μm; *N* = 3 mice/group, four fields/mouse; representative fields are shown). Ratios of the levels of these factors in all groups relative to levels in the Kr-DN (4w) group are also included. Data graphed denote the mean ± SD of the mean (SD). Statistical significance was determined using one-way ANOVA followed by Tukey test. *, 0.01 < *P* < 0.05; **, 0.001 < *P* < 0.01; ***, 0.0001 < *P* < 0.001; ****, *P* < 0.0001.

We evaluated the effects of ectopic BMI1 overexpression on SC proliferation using IHC (antibodies in Supplementary Table S3). As expected, BMI1 protein was expressed in most basal SCs and some suprabasal cells of tongue epithelia from all experimental groups listed in Fig. 1B, and BMI1 levels were increased in KrTB-DN (4w) versus Kr-DN (4w) and KrTB-DN (10w) versus Kr-DN (10w) (Fig. 2A). We further verified that BMI1 is overexpressed in KrTB-DN compared with Kr-DN groups via qPCR, using RNA isolated from tongue epithelia (Supplementary Fig. S4; Supplementary Table S4). We then analyzed the expression of Ki67, a marker of actively proliferating cells (41), such as the basal SCs of oral epithelia (29). IHC showed that Ki67 expression increased 1.3-fold (*P* = 0.012) in KrTB-DN (4w) versus Kr-DN (4w) and 1.2-fold (*P* = 0.037) in KrTB-DN (10w) versus Kr-DN (10w) (Fig. 2B).

Additionally, we assessed oxidative stress by measuring 4-hydroxynonenal (4-HNE) levels in all experimental groups listed in Fig. 1B. 4-HNE is a marker of oxidative stress resulting from the accumulation of ROS (42). 4-HNE levels were 5.2-fold higher (*P* < 0.0001) in KrTB-DN (4w) compared with Kr-DN (4w), as well 1.9-fold higher (*P* = 0.0001) in KrTB-DN (10w) compared with Kr-DN (10w) tongue epithelia (Fig. 2C). Overall, our RNA-seq and IHC data indicate that ectopic BMI1 overexpression further increases proliferation and oxidative stress in tongue epithelia as early as 4 weeks after initiation of 4-NQO treatment.

### Increases in OSCC- and glycolysis-associated factors occur early in 4-NQO–induced tumorigenesis upon BMI1 overexpression

**Figure 3.**
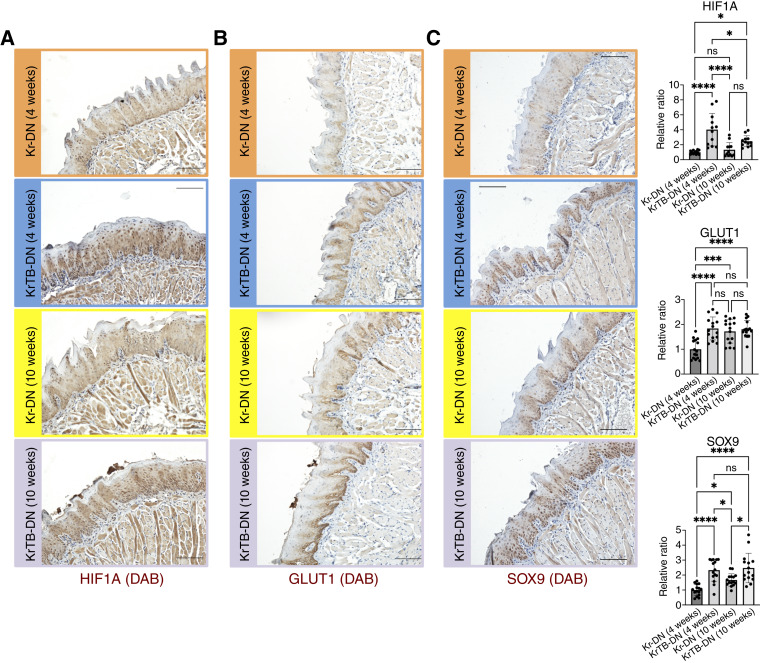
Increases in OSCC biomarkers occur early in 4-NQO–induced tumorigenesis upon BMI1 overexpression. **A,** HIF1α, (**B**) GLUT1, and (**C**) SOX9 IHC stainings in Kr-DN (4w), KrTB-DN (4w), Kr-DN (10w), and KrTB-DN (10w) tongue epithelia (200×; scale bar, 100 μm; *N* = 3 mice/group, four fields/mouse; representative fields are shown). Ratios of the levels of these factors in all groups relative to levels in the Kr-DN (4w) group are also included. Data graphed denotes the mean ± SD of the mean (SD). Statistical significance was determined using one-way ANOVA followed by Tukey test. *, 0.01 < *P* < 0.05; ***, 0.0001 < *P* < 0.001; ****, *P* < 0.0001.

Our RNA-seq analyses comparing KrTB-DN (4w) and KrTB-DN (10w) groups to the control KrTB-N (25w) group also revealed other transcripts associated with human OSCC (36) that are increased at 4 and 10 weeks upon BMI1 overexpression (Supplementary Fig. S5). These transcripts include SOX9, matrix metalloproteinase-9 (MMP9), CAR9, HIF1A, SLC16A3 (MCT4), and SLC2A1 (GLUT1; Supplementary Fig. S5), which all have been linked to poor OSCC patient survival (31, 43–47). We then performed IHC (antibodies in Supplementary Table S3) to quantify the protein levels of some of these OSCC biomarkers in all experimental groups from Fig. 1B. These IHC data included HIF1α (Fig. 3A), GLUT1 (SLC2A1; Fig. 3B), and SOX9 (Fig. 3C). Indeed, HIF1α (4.0 fold, *P* < 0.0001), GLUT1 (1.8 fold, *P* < 0.0001), and SOX9 (2.3 fold, *P* < 0.0001) levels were increased in the KrTB-DN (4w) versus Kr-DN (4w) group (Fig. 3).

**Figure 4.**
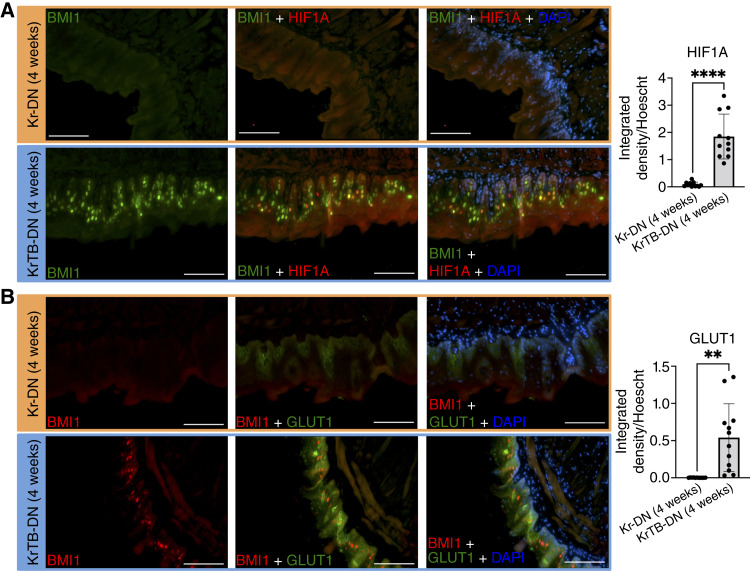
Cells with ectopic BMI1 and increased HIF1α or GLUT1 expression are detected in 4-NQO–treated tongue epithelia at an early time point (4 weeks). **A,** BMI1 and HIF1α IF stainings in samples from Kr-DN (4w) and KrTB-DN (4w) tongue epithelia. **B,** BMI1 and GLUT1 IF stainings in samples from Kr-DN (4w) and KrTB-DN (4w) tongue epithelia. For **A** and **B**, we report ratios of integrated density of HIF1α or GLUT1 (measured by Fiji) over integrated density of Hoechst signal in the same area (*N* = 3 mice/group, four fields/mouse). Representative fields are shown (200×; scale bar, 100 μm). All data graphed denote the mean ± SD of the mean (SD). Statistical significance was determined using Welch *t* test. **, 0.001 < *P* < 0.01; ****, *P* < 0.0001.

As the early increases in these OSCC biomarkers (Fig. 3) correlated with higher percentages of mice with hyperplasia/dysplasia in the KrTB-DN (4w) versus the Kr-DN (4w) groups (Fig. 1D), we examined these groups using IF. To assess the colocalization of BMI1 with some of these targets, we stained for BMI1 and either HIF1α (Fig. 4A) or GLUT1 (Fig. 4B) proteins in samples from KrTB-DN (4w) and Kr-DN (4w) groups (antibodies in Supplementary Table S3). IF confirmed that KrTB-DN (4w) epithelia contained BMI1-overexpressing cells with high HIF1α or GLUT1 expression. In contrast, Kr-DN (4w) epithelia did not display any BMI1+ cells with high HIF1α or GLUT1 expression, as expected (Fig. 4). We verified increased HIF1A and SLC2A1 (GLUT1) mRNA expression in the KrTB-DN (4w) compared with the Kr-DN (4w) group via qRT-PCR, using RNA isolated from tongue epithelia (Supplementary Fig. S6; Supplementary Table S4). Collectively, our IF and qPCR data demonstrate that these early increases in HIF1α or GLUT1 result from exogenous BMI1 overexpression.

Lastly, examination of the OSCC-associated targets that are increased earlier upon BMI1 overexpression revealed transcripts involved in glycolysis, such as SLC2A1 and SLC16A3 (Supplementary Fig. S5). Other metabolic targets, which are also HIF1α downstream targets (48, 49), were further increased in KrTB-DN (4w) and KrTB-DN (10w) compared with the KrTB-N (25w) group (Supplementary Fig. S7). Some of these targets include PKM, HK2, and TPI1, which encode for key enzymes in glycolysis, as well as LDHA, which encodes for the enzyme that converts pyruvate into lactate after glycolysis (50). We also assessed the protein levels of some of these HIF1α downstream targets, including SLC16A3 (Supplementary Fig. S8A), PKM2 (Supplementary Fig. S8B), and GPI1 (Supplementary Fig. S8C), in the experimental groups from Fig. 1B. As expected, all of these proteins were also increased in the KrTB-DN (4w) versus the Kr-DN (4w) group (Supplementary Fig. S8). Taken together, these results demonstrate that BMI1 overexpression accelerates these increases in OSCC biomarkers during 4-NQO treatment, including some HIF1α downstream targets associated with glycolysis.

### BMI1 KO in the human OSCC line SCC-25 decreases the expression of HIF1α and glycolysis-associated protein GLUT1

We extended our studies to a human cell model of OSCC (SCC-25) in which we can further elucidate the molecular mechanisms of BMI1 during the disease. As HIF1α is an early downstream target of BMI1 in our 4-NQO model (Figs. 3 and 4), and BMI1 associates with the promoter of the HIF1A gene in mouse tongue epithelia (26), we first assessed whether BMI1 also associated with the promoter region of the HIF1A gene in SCC-25 cells through two-step ChIP. Using compiled genome-wide ChIP sequencing data from the UCSC Genome Browser, we designed HIF1A primers to amplify promoter regions bound by multiple transcription factors (Supplementary Fig. S9A; Supplementary Table S5). Our ChIP-PCR data confirmed that BMI1 associated with these regions in the HIF1A promoter of SCC-25 cells (Supplementary Fig. S9B and S9C). We validated these results by verifying the association of BMI1 with the PTEN promoter (Supplementary Fig. S9D), an established BMI1 downstream target (51), as well as with the use of negative control primers designed around the HPRT1 gene (Supplementary Fig. S9E).

**Figure 5.**
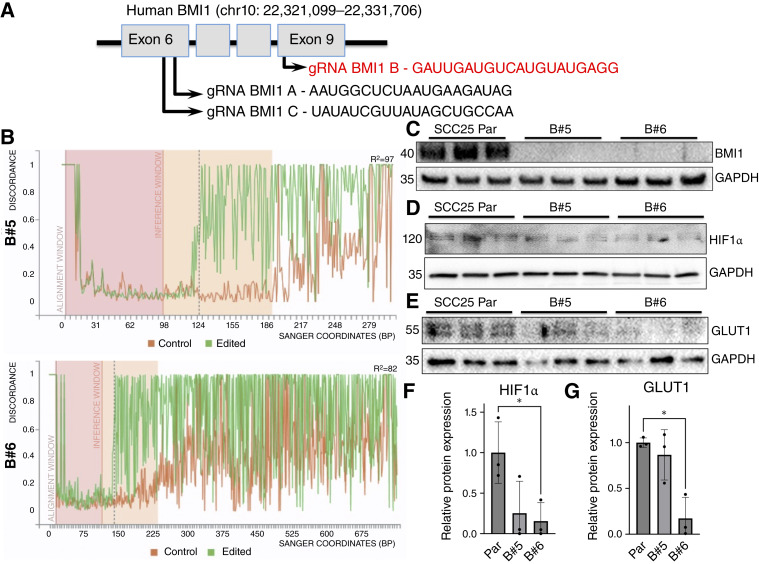
BMI1 KO in the human OSCC line SCC-25 decreases the expression of HIF1α and glycolysis-associated protein GLUT1. **A,** A scheme of Crispr/Cas9 technology strategy indicating the three gRNAs designed in exons 6 and 9 of the human BMI1 gene. Treatment with gRNA B (in red) resulted in the highest percentage of editing. **B,** Discordance plots detailing the level of alignment per base between the parental (control) and the edited (B#5 or B#6) SCC-25 cell lines in the interference windows, based on Sanger sequencing of fragments amplified via PCR from genomic DNA of treated cells. On the plots, green and orange lines are close together before the cutsite (dashed line) and remain far apart after this site. These plots were generated using the online tool provided by Synthego, ICE. Immunoblotting of the protein levels of (**C**) BMI1 and GAPDH, (**D**) HIF1α and GAPDH, and (**E**) GLUT1 and GAPDH in parental, B#5, and B#6 SCC-25 cells (triplicates). Quantifications of (**F**) HIF1α and (**G**) GLUT1 immunoblottings (relative to GAPDH) are included. Statistical significance was determined relative to parental SCC-25 cells using Welch *t* test (*, 0.01 < *P* < 0.05).

Next, we generated BMI1 KO SCC-25 cells using CRISPR/Cas9 technology (Fig. 5A, gRNAs in Supplementary Table S6). qPCR using primers designed around expected edited sites (Supplementary Table S7) revealed the gRNA (BMI1 B) that derived the highest percentage of edited cells (Fig. 5A). gRNA BMI1 B targets a location in exon 9 that encodes for the helix-turn-helix motif in the BMI1 protein, a motif upstream of the nuclear localization signal #2 (NLS2) required for the binding of the BMI1 protein to DNA (52). Sanger sequencing (Fig. 5B) showed that B#6 had the highest KO score (proportion of cells with frameshift-inducing indels) out of the two resulting BMI1 KO lines (Supplementary Fig. S10). Using Parental (Par) SCC-25 cells incubated with lipofectamine only as controls, we then confirmed that the BMI1 protein is not expressed in the B#5 and B#6 SCC-25 cell lines (Fig. 5C, antibodies in Supplementary Table S8).

As our RNA-seq, IHC, and IF data in the KrTB mouse model showed that BMI1 overexpression accelerates the increase in OSCC markers, including glycolysis targets, in 4-NQO–treated tongue epithelia (Figs. 3 and 4; Supplementary Fig. S5–S8), we assessed the expression of some of these targets in BMI1 KO SCC-25 cells. Whereas HIF1α and GLUT1 expression was reduced in both BMI1 KO lines, these decreases were significant in B#6 cells (Fig. 5D–G, antibodies in Supplementary Table S8), the BMI1 KO line with the highest knockout score (Supplementary Fig. S10). Thus, BMI1 deletion in SCC-25 cells decreases the expression of HIF1α and the glycolysis-associated protein GLUT1, early human OSCC biomarkers (53–56) that are also elevated in the early stages of 4-NQO induced tumorigenesis upon BMI1 overexpression.

### Proliferation and oxidative stress are decreased in the human OSCC line SCC-25 upon BMI1 deletion

**Figure 6.**
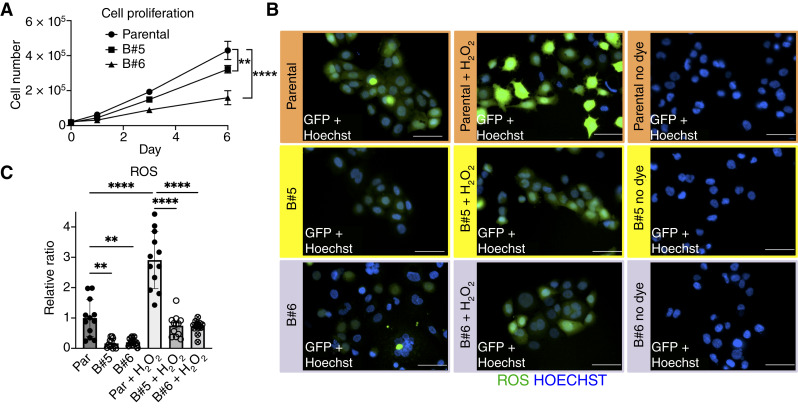
Proliferation and oxidative stress are decreased in the human OSCC line SCC-25 upon BMI1 deletion. **A,** Proliferation in parental, B#5, and B#6 SCC25 cells using a cell counter (*N* = 6 replicates/time point). Data graphed denotes the mean ± SD of the mean (SD). Statistical significance was determined using two-way RM ANOVA followed by Tukey multiple comparisons test. Significance values shown were calculated relative to the parental group. **B,** ROS in parental, B#5, and B#6 SCC25 cells, with and without H_2_O_2_ induction. Representative images are shown for each of the six experimental groups and the three negative (no dye) controls. All images are 200×; scale bar, 100 μm. **C,** Quantification of ROS data from **B**, with *N* = 3 replicates/group, four fields/group. Data graphed denotes the mean ± SD of the mean (SD). Statistical significance was determined using one-way ANOVA followed by Tukey test. Significance was calculated relative to the parental (no H_2_O_2_) group. For **A** and **C**, **, 0.001 < *P* < 0.01; ****, *P* < 0.0001.

We also examined the effects of BMI1 deletion on proliferation and oxidative stress of SCC-25 cells, as we observed early increases in these two processes in 4-NQO–treated mice upon BMI1 overexpression (Fig. 2; Supplementary Figs. S2 and S3). We detected decreased proliferation in both B#5 and B#6 cell lines compared with parental SCC-25 cells (Fig. 6A). This decrease is more pronounced in B#6 cells, which also exhibited the highest KO score of the two BMI1 KO lines (Supplementary Fig. S10). Next, we loaded Parental, B#5, and B#6 SCC-25 cells with CM-H_2_DCFDA to define the effects of a BMI1 KO on the ROS levels of these human OSCC cells. A product of the oxidation of CM-H_2_DCFDA, DCF is highly fluorescent compound that indicates ROS production (26, 57). Our data show that ROS levels are decreased in B#5 and B#6 compared with parental SCC-25 cells (Fig. 6B and C). This decrease in ROS levels was also detected upon BMI1 deletion when all cell lines were incubated with 100 μmol/L H_2_O_2_ to induce oxidative activity (Fig. 6B and C). Hence, BMI1 deletion also decreases oxidative stress in SCC-25 cells.

Taken together, the results in SCC25 cells demonstrate that BMI1 deletion in a human OSCC line can decrease proliferation and oxidative stress levels, as well as reduce the expression of HIF1α and a HIF1α downstream target linked to glycolysis, GLUT1. Thus, by deleting BMI1 in this human oral squamous cell carcinoma (OSCC) model, we confirmed that BMI1 increases multiple markers early in 4-NQO–induced tumorigenesis (Supplementary Fig. S11).

## Discussion

### Increased proliferation in tongue epithelia is an early OSCC event that is accelerated by BMI1

We previously demonstrated that exogenous BMI1 overexpression in basal epithelial SCs enhanced tumor formation upon 4-NQO treatment in our OSCC mouse model (24). Furthermore, BMI1 expression exacerbated OSCCs by increasing the percentage of invasive lesions and promoting glycolysis during late stages (25). In this study, we identify early OSSC-associated markers that are accelerated upon BMI1 overexpression in our 4-NQO mouse model. Our data show that BMI1 overexpression increases basal layer SC proliferation in tongue epithelia during 4-NQO–induced carcinogenesis (Fig. 2B; Supplementary Fig. S2). This increase is detected as early as 4 weeks in the tumorigenesis process (Fig. 2B). In line with this observation, cell proliferation is reduced in the SCC-25 cell line when BMI1 is deleted (Fig. 6A).

Other studies analyzing samples from human patients with OSCCs showed that increased BMI1 expression was detected early during oral carcinogenesis (18, 23). For instance, oral leukoplakia and inflammatory hyperplasia specimens (premalignant lesions) displayed higher BMI1 expression compared with normal oral mucosa samples (23). Furthermore, BMI1 expression gradually increased over time and directly correlated with Ki67 expression during OSCC progression (23). Ki67, a marker of actively proliferating cells, is highly expressed in the basal SCs of oral epithelia (29, 41). Ki67-positive cells were decreased when labeled BMI1-positive SCs were eliminated in murine tongue epithelia through the use of cesium irradiation (58), providing more evidence for a direct correlation between BMI1 and Ki67 in basal epithelia SCs, even before tumorigenesis. We also previously detected increased Ki67 expression in BMI1-overexpressing mouse tongue epithelia under *normal* conditions (26). Thus, our data presented here suggest that SC proliferation in tongue epithelia is regulated by BMI1 before and during the early stages of oral carcinogenesis.

Studies in other human OSCC cell lines and xenograft models have also indicated that cell proliferation is impaired when BMI1 is targeted with the selective BMI1 inhibitor PTC-209 (59). PTC-209 treatment resulted in G1-phase cell-cycle arrest in two HNSCC cell lines (Cal27 and FaDu), as well as significantly reduced tumor growth in a xenograft model using these cells (59).

In this study, we detected *decreased* proliferation upon BMI1 KO in the human OSCC cell line (SCC-25 cells, Fig. 6A). Also, our laboratory previously established increased cell-cycle progression, as well as increased expression of targets involved in DNA replication, as early events in 4-NQO–induced carcinogenesis (36). Some of these targets included Aurora kinase B, cyclins B1 and F, cyclin-dependent kinase 1, and Minichromosome maintenance complex members 5 and 10 (36). Our RNA-seq data in mice show that most of these targets are further increased with ectopic BMI1 as early as 4 weeks in the tumorigenesis process and continue to increase at 10 weeks of 4-NQO treatment (Supplementary Fig. S2). More studies are needed to fully characterize how specific members of the cell-cycle regulatory network, which are activated during the early stages of tongue tumorigenesis, are regulated by BMI1, ultimately resulting in elevated Ki67 levels and increased cell proliferation.

### Expression of OSCC and glycolysis biomarkers is further increased during tumorigenesis when ectopic BMI1 is overexpressed

Examples of OSCC targets that show greater increases upon ectopic BMI1 overexpression during 4-NQO treatment include SOX9, MMP9, CXCL5, CD274, and CAR9 (Supplementary Fig. S5A; ref. 36). SOX9 promoted stemness and epithelial-to-mesenchymal transition in an *in vitro* HNSCC model (60), and high SOX9 protein expression in patients with OSCC correlated with poorer clinical outcomes and radioresistance (61). We previously reported that SOX9 protein is detected, together with BMI1, in the infiltrating cells of invasive SCCs (25). In this study, we demonstrate that SOX9 protein expression is accelerated with ectopic BMI1 during early tumorigenesis (Fig. 3B). Levels of MMP9 protein are higher in saliva of patients with oral cancer compared with healthy controls, and this could be a potential biomarker for OSCC early detection (47). Chemokine CXCL5 plays roles in inflammation and immune responses and is elevated in extracellular vesicles isolated from OSCC cell lines (62). PD-L1 (gene: CD274) is also elevated in patients with HNSCC, and its expression is associated with cancer chemoresistance (63). Finally, transmembrane enzyme carbonic anhydrase IX (CAR9/CAIX) is a tumor prognostic marker in patients with OSCC (64). Knockdown of CAR9 inhibits OSCC cell proliferation and migration *in vitro* (65). Thus, these BMI1 downstream targets are likely biomarkers for early OSCC detection and treatment.

Multiple metabolic targets are part of a particular subset of OSCC-associated mRNAs that are increased early with ectopic BMI1 overexpression during 4-NQO induced carcinogenesis. This group includes transcripts encoding glycolytic enzymes such as MCT4 (SLC16A3, Supplementary Fig. S5A), LDHA, PKM, GPI1, PFKL, and HK2, among others (Supplementary Fig. S7A). All of these targets contribute to metabolic reprogramming in tumors, primarily favoring glycolysis over oxidative phosphorylation even under normoxic conditions, in what is known as the Warburg effect (7, 9, 10, 50). Metabolic reprogramming provides the energy to support rapid tumor progression during HNSCC through increased glucose breakdown (66). Evidence for metabolic reprogramming had been observed in mouse samples collected at late stages of oral carcinogenesis, when lesions become invasive (36). We also demonstrated that ectopic BMI1 expression enhanced 4-NQO–induced tumorigenesis by promoting glycolysis during the late stages of the disease (25). In this study, our data suggest increased metabolic reprogramming and elevated glycolysis upon BMI1 overexpression in the early stages of the disease (Supplementary Figs. S7 and S8), before lesions are visible (4 weeks of 4-NQO). Other studies conducted in human patient samples also demonstrated an early shift to favor glycolytic pathways, including in precancerous lesions (53, 67). Thus, our data suggest that metabolic reprogramming in tongue epithelia, a key feature of invasive lesions (25), is also increased by high BMI1 expression during the early stages of oral carcinogenesis.

Some of these metabolic targets contain hypoxia-responsive elements that are bound and regulated by HIF1α (49, 50). In our SCC-25 OSCC model, we confirmed that the expression of HIF1α protein and its downstream target GLUT1 (SLC2A1) is greatly reduced in the SCC-25 cells (B#6 line) with the highest BMI1 KO score (Fig. 5). Likewise, increases in these two proteins are accelerated during 4-NQO–induced tumorigenesis with ectopic BMI1 overexpression (Figs. 3 and 4). HIF1α is a key transcription factor involved in energy metabolism and invasion potential of OSCC cells (45, 49), and more than 90% of samples of metastatic lymph nodes from patients with OSCC contained cells with high HIF1α expression (68). Moderate and severe HIF1α staining intensity was detected in human samples of oral submucous fibrosis, a precancerous condition (55). Similarly, GLUT1 is the primary glucose transporter that regulates the rate of glycolysis, and its high expression correlates with poorer survival rates in patients with HNSCC (44). Increased GLUT1 protein expression was also detected in human samples of early epithelial dysplasia (53). We previously reported that GLUT1 protein is detected in the infiltrating cells of invasive SCCs (25). Additionally, BMI1 associates with the promoter of the HIF1A gene in SCC-25 cells (Supplementary Fig. S9) and in mouse tongue epithelia (26). Thus, we propose that the BMI1-induced increase of metabolic targets, observed as early as at 4 weeks in our 4-NQO model, possibly involves HIF1α as an intermediate transcription factor. Further studies are necessary to determine whether BMI1 can also bind directly on glycolytic genes, such as SLC2A1 (GLUT1) or SLC16A3, or genes encoding other OSCC biomarkers that, in addition to HIF1α, regulate the expression of downstream metabolic targets.

### Oxidative stress is also increased early during tumorigenesis when BMI1 is overexpressed

Our data show that BMI1 overexpression also increases oxidative stress in tongue epithelia during 4-NQO–induced carcinogenesis, and this increase is detected as early as at 4 weeks of 4-NQO treatment (Fig. 2C). Oxidative stress is a major process in the oral mucosal disease pathogenesis, starting early and becoming more severe through malignant transformation (69). Indeed, other reports have provided additional evidence of increased oxidative stress, such as higher levels of markers of lipid peroxidation and reduced superoxide dismutase expression, in the early stages of OSCC, including in leukoplakia and other premalignant lesions (70). Substantial redox imbalance is common in early-stage OSCC, with studies suggesting that some of the oxidative stress markers, such as elevated malondialdehyde and nitric oxide in patient blood samples, as well as decreases in reduced glutathione in saliva, could be used as potential biomarkers in early OSCC diagnosis (71, 72). Our laboratory also established increased response to oxidative stress, as well as increased metabolic response to ROS, as early events in carcinogen-induced tumorigenesis in our 4-NQO mouse model (36). Thus, our findings connecting BMI1 overexpression to increased oxidative stress during early-stage OSCC demonstrate that BMI1 accelerates another important feature of human oral cancers.

We also demonstrate that ROS levels, which cause oxidative stress in cells (42), are reduced in the complementary SCC-25 cell model when BMI1 is deleted (Fig. 6B and C). These data confirmed that BMI1 promotes oxidative stress, a key feature in OSCC. Increased oxidative stress has been observed in other human OSCC cell culture models, such as HSC-3, SCC9, and Ca9.22 lines (73). Our RNA-seq data show that high BMI1 expression decreases the expression of PRDX5 in KrTB-DN (4w) versus KrTB-N (25w) (Supplementary Fig. S3A), and PXDX5, Catalase (CAT), and PRDX2 in KrTB-DN (10w) versus KrTB-N (25w) (Supplementary Fig. S3B). As these transcripts are involved in reducing ROS (9, 74) and BMI1 is a member of the PRC1 (21), our findings suggest transcriptional repression of gene expression by BMI1. Other studies in CSCs showed that changes in BMI1 expression result in mitochondrial dysfunction (75), which is observed in early-stage oral lesions and can lead to increased ROS and genomic instability (76).

### Conclusions

We conclude that BMI1 overexpression accelerates key, early-stage events in 4-NQO–induced tumorigenesis, resulting in more oncogenic changes in mice (Supplementary Fig. S11). These markers of tumorigenesis include increased proliferation and oxidative stress, as well as increased expression of multiple transcripts and proteins linked to human OSCCs, such as HIF1A and GLUT1 (SLC2A1), in murine tongue epithelia during 4-NQO–induced carcinogenesis. Several of these OSCC biomarkers increased by ectopic BMI1 expression, including SLC16A, LDHA, PKM2, and GPI1, have also been linked to metabolic reprogramming that occurs in precancerous lesions and during oral carcinogenesis (53, 67). In a human OSCC cell line (SCC-25) with BMI1 knocked out, a complementary model, we observed decreases in proliferation, oxidative stress, and expression of HIF1α and GLUT1 proteins (Supplementary Fig. S11). Thus, inhibition of BMI1 could be a potential target for cancer prevention approaches and merits further consideration and additional functional studies.

## Supplementary Material

Supplementary Table 1 Differentially expressed genes in tongue epithelia of KrTB-DN (4w) vs. KrTB-N (25w) mice (Excel File)

Supplementary Table 2 Differentially expressed genes in tongue epithelia of KrTB-DN (10w) vs. KrTB-N (25w) mice (Excel File)

Supplementary Figure 1 Human OSCC-associated genes that are further increased in KrTB-DN (10w) compared to KrTB-DN (4w) tongue epithelia.

Supplementary Figure 2 Genes involved in cell cycle regulation and DNA replication that are further increased in 4-NQO-treated tongue epithelia at early times points upon BMI1 overexpression.

Supplementary Figure 3 Genes involved in ROS regulation that are differentially expressed in 4-NQO-treated tongue epithelia at early times points upon BMI1 overexpression.

Supplementary Figure 4 BMI1 mRNA levels are increased in KrTB-DN vs. Kr-DN tongue epithelia after 4 or 10 weeks of 4-NQO treatment.

Supplementary Figure 5 Human OSCC-associated genes that are further increased in 4-NQO-treated tongue epithelia at early times points upon BMI1 overexpression.

Supplementary Figure 6 BMI1 overexpression in tongue epithelia increases HIF1A and SLC2A1 mRNA levels after 4 weeks of 4-NQO treatment.

Supplementary Figure 7 Metabolic targets that are increased in 4-NQO-treated tongue epithelia at early times points and upon BMI1 overexpression.

Supplementary Figure 8 Increases in glycolysis-associated factors occur early in 4-NQO-induced tumorigenesis upon BMI1 overexpression.

Supplementary Figure 9 BMI1 associates with the promoter region of the HIF1A gene in the human OSCC line SCC-25.

Supplementary Figure 10 Relative contributions (%) of inferred sequences detected in B#5 and B#6 SCC-25 cells, compared to Parental SCC-25 cells.

Supplementary Figure 11 Key changes regulated by BMI1 in two OSCC models.

Supplementary Table 3 Antibody list for Immunofluorescence/ Immunohistochemistry

Supplementary Table 4 List of primers used in qRT-PCR (Mouse Tissues)

Supplementary Table 5 List of primers used in ChIP-qPCR (SCC-25 cells)

Supplementary Table 6 guide RNA (gRNA) sequences used to knock out BMI1 in SCC-25 cells

Supplementary Table 7 Primers used to amplify regions around expected edited sites (via PCR) for gRNAs employed in BMI1 knockouts (KO)

Supplementary Table 8 Antibody list for Western Blotting

## Data Availability

The RNA-seq data have been deposited in the GEO (NCBI) repository (accession number GSE307391) and are publicly accessible. All other data needed to evaluate the conclusions in the article are available in the main text or the supplementary materials.
