# Supplementary material for: Key Early Changes in Oral Squamous Cell Carcinogenesis Are Accelerated by Ectopic BMI1 Expression
Source: Cancer Res Commun. 2026 Jan 20;6(1):152–64. doi: 10.1158/2767-9764.CRC-25-0580 (PMC12816948; doi:10.1158/2767-9764.CRC-25-0580)
Supplement: Supplementary Figure 1 — Human OSCC-associated genes that are further increased in KrTB-DN (10w) compared to KrTB-DN (4w) tongue epithelia. [file crc-25-0580_supplementary_figure_1_suppsf1.docx]

**Supplementary Figure 1.** Human OSCC-associated genes that are further increased in KrTB-DN (10w) compared to KrTB-DN (4w) tongue epithelia. (**A**) Timeline outlining the different experimental groups used in RNA sequencing analyses. Samples were collected at ages 10, 16, or 31 weeks old (4-NQO treatment for 4 or 10 weeks, respectively; or 15 weeks post 10 weeks of 4-NQO treatment). (**B**) Fold change of gene expression levels of OSCC-associated genes in KrTB-DN (10w) vs. KrTB-N (4w) tongue epithelia. Data graphed denotes the mean ± standard deviation of the mean (SD).
