# Supplementary material for: Key Early Changes in Oral Squamous Cell Carcinogenesis Are Accelerated by Ectopic BMI1 Expression
Source: Cancer Res Commun. 2026 Jan 20;6(1):152–64. doi: 10.1158/2767-9764.CRC-25-0580 (PMC12816948; doi:10.1158/2767-9764.CRC-25-0580)
Supplement: Supplementary Figure 2 — Genes involved in cell cycle regulation and DNA replication that are further increased in 4-NQO-treated tongue epithelia at early times points upon BMI1 overexpression. [file crc-25-0580_supplementary_figure_2_suppsf2.docx]

**Supplementary Figure 2.** Genes involved in cell cycle regulation and DNA replication that are further increased in 4-NQO-treated tongue epithelia at early times points upon BMI1 overexpression. (**A**) Fold change of gene expression levels of cell cycle/DNA replication targets in KrTB-DN (4w) vs. KrTB-N (25w) tongue epithelia. (**B**) Fold change of gene expression levels of cell cycle/DNA replication targets in KrTB-DN (10w) vs. KrTB-N (25w) tongue epithelia. All data graphed denotes the mean ± standard deviation of the mean (SD).
