# Supplementary material for: Key Early Changes in Oral Squamous Cell Carcinogenesis Are Accelerated by Ectopic BMI1 Expression
Source: Cancer Res Commun. 2026 Jan 20;6(1):152–64. doi: 10.1158/2767-9764.CRC-25-0580 (PMC12816948; doi:10.1158/2767-9764.CRC-25-0580)
Supplement: Supplementary Figure 4 — BMI1 mRNA levels are increased in KrTB-DN vs. Kr-DN tongue epithelia after 4 or 10 weeks of 4-NQO treatment. [file crc-25-0580_supplementary_figure_4_suppsf4.docx]

**Supplementary Figure 4.** BMI1 mRNA levels are increased in KrTB-DN vs. Kr-DN tongue epithelia after 4 or 10 weeks of 4-NQO treatment. Tongue epithelia from (**A**) Kr-DN (4w) and KrTB-DN (4w), and (**B**) Kr-DN (10w) and KrTB-DN (10w) mice were separated and homogenized (*N* = 3-7 mice/group). Isolated RNA samples were reverse-transcribed and subjected to qRT-PCR analysis to measure mRNA expression levels of BMI1 compared to 36B4 (control). Ratios of BMI1 mRNA levels relative to levels in the Kr-DN (4w) and Kr-DN (10w) groups are depicted for (A) and (B), respectively. All data graphed denotes the mean ± standard deviation of the mean (SD). Statistical significance was determined using Welch’s t-test, *0.01<p<0.05, **0.001<p<0.01.
