# Supplementary material for: Key Early Changes in Oral Squamous Cell Carcinogenesis Are Accelerated by Ectopic BMI1 Expression
Source: Cancer Res Commun. 2026 Jan 20;6(1):152–64. doi: 10.1158/2767-9764.CRC-25-0580 (PMC12816948; doi:10.1158/2767-9764.CRC-25-0580)
Supplement: Supplementary Figure 6 — BMI1 overexpression in tongue epithelia increases HIF1A and SLC2A1 mRNA levels after 4 weeks of 4-NQO treatment. [file crc-25-0580_supplementary_figure_6_suppsf6.docx]

**Supplementary Figure 6.** BMI1 overexpression in tongue epithelia increases HIF1A and SLC2A1 mRNA levels after 4 weeks of 4-NQO treatment. Tongue epithelia from Kr-DN (4w) and KrTB-DN (4w) mice were separated and homogenized (*N* = 3-9 mice/group). Isolated RNA samples were reverse-transcribed and subjected to qRT-PCR analysis to measure mRNA expression levels of (**A**) HIF1A and (**B**) SLC2A1 (GLUT1) compared to 36B4 (control). Ratios of mRNA levels relative to levels in the Kr-DN (4w) group are depicted. All data graphed denotes the mean ± standard deviation of the mean (SD). Statistical significance was determined using Welch’s t-test, *0.01<p<0.05, **0.001<p<0.01.
