# Supplementary material for: Key Early Changes in Oral Squamous Cell Carcinogenesis Are Accelerated by Ectopic BMI1 Expression
Source: Cancer Res Commun. 2026 Jan 20;6(1):152–64. doi: 10.1158/2767-9764.CRC-25-0580 (PMC12816948; doi:10.1158/2767-9764.CRC-25-0580)
Supplement: Supplementary Figure 7 — Metabolic targets that are increased in 4-NQO-treated tongue epithelia at early times points and upon BMI1 overexpression. [file crc-25-0580_supplementary_figure_7_suppsf7.docx]

**Supplementary Figure 7.** Metabolic targets that are increased in 4-NQO-treated tongue epithelia at early times points and upon BMI1 overexpression. (**A**) Fold change of gene expression levels of metabolic targets in KrTB-DN (4w) vs. KrTB-N (25w) tongue epithelia. (**B**) Fold change of gene expression levels of metabolic targets in KrTB-DN (10w) vs. KrTB-N (25w) tongue epithelia. All data graphed denotes the mean ± standard deviation of the mean (SD).
