# Supplementary material for: Key Early Changes in Oral Squamous Cell Carcinogenesis Are Accelerated by Ectopic BMI1 Expression
Source: Cancer Res Commun. 2026 Jan 20;6(1):152–64. doi: 10.1158/2767-9764.CRC-25-0580 (PMC12816948; doi:10.1158/2767-9764.CRC-25-0580)
Supplement: Supplementary Figure 8 — Increases in glycolysis-associated factors occur early in 4-NQO-induced tumorigenesis upon BMI1 overexpression. [file crc-25-0580_supplementary_figure_8_suppsf8.docx]

**Supplementary Figure 8.** Increases in glycolysis-associated factors occur early in 4-NQO-induced tumorigenesis upon BMI1 overexpression. (**A**) SLC16A3, (**B**) PKM2, and (**C**) GPI1 IHC stainings in Kr-DN (4w), KrTB-DN (4w), Kr-DN (10w), and KrTB-DN (10w) tongue epithelia (200X; scale bar: 100 μm; *N* = 3 mice/group, 4 fields/mouse; representative fields are shown). Ratios of the levels of these factors in all groups relative to levels in the Kr-DN (4w) group are also included. Data graphed denotes the mean ± standard deviation of the mean (SD). Statistical significance was determined using one-way ANOVA followed by Turkey’s test. *0.01<p<0.05, **0.001<p<0.01, ***0.0001<p<0.001, ****p<0.0001.
