# Supplementary material for: Key Early Changes in Oral Squamous Cell Carcinogenesis Are Accelerated by Ectopic BMI1 Expression
Source: Cancer Res Commun. 2026 Jan 20;6(1):152–64. doi: 10.1158/2767-9764.CRC-25-0580 (PMC12816948; doi:10.1158/2767-9764.CRC-25-0580)
Supplement: Supplementary Figure 9 — BMI1 associates with the promoter region of the HIF1A gene in the human OSCC line SCC-25. [file crc-25-0580_supplementary_figure_9_suppsf9.docx]

**Supplementary Figure 9.** BMI1 associates with the promoter region of the HIF1A gene in the human OSCC line SCC-25. Replicates of SCC-25 cells were isolated and homogenized, and the resulting soluble chromatin samples (10 μg) were immunoprecipitated (IP’ed) with 1 μg of antibodies specific for BMI1 (*N* = 4) or IgG (negative control, *N* = 4). Purified DNA was then used in qPCR analyses. (**A**) Schematic representation of the HIF1A gene structure that shows the region used to design primers for ChIP-qPCR. Primers used in qPCR reactions were specific for (**B**) HIF1A promoter (Primers 1), (**C**) HIF1A promoter (Primers 4), (**D**) PTEN promoter (positive control), and (**E**) a region in the HPRT1 gene (negative control). Binding is expressed relative to the pre-IP input DNA. Data graphed denotes the mean ± standard deviation of the mean (SD). Statistical significance was determined using Welch’s t-test. *0.01<p<0.05, **0.001<p<0.01
