# Supplementary material for: Key Early Changes in Oral Squamous Cell Carcinogenesis Are Accelerated by Ectopic BMI1 Expression
Source: Cancer Res Commun. 2026 Jan 20;6(1):152–64. doi: 10.1158/2767-9764.CRC-25-0580 (PMC12816948; doi:10.1158/2767-9764.CRC-25-0580)
Supplement: Supplementary Figure 10 — Relative contributions (%) of inferred sequences detected in B#5 and B#6 SCC-25 cells, compared to Parental SCC-25 cells. [file crc-25-0580_supplementary_figure_10_suppsf10.docx]

**Supplementary Figure 10.** Relative contributions (%) of inferred sequences detected in (**A**) B#5 and (**B**) B#6 SCC-25 cells, compared to Parental SCC-25 cells. These results are based on Sanger sequencing of fragments amplified from genomic DNA via PCR. Cut sites are represented by black vertical dotted lines. Indel percentages indicate the proportion of cells with edits (i.e. editing efficiency). Knockout-Scores reveal the proportion of cells with frameshift-inducing indels. These images were generated using the online tool provided by Synthego (Redwood City, CA), Inference of CRISPR Edits (ICE).
