# Supplementary material for: Key Early Changes in Oral Squamous Cell Carcinogenesis Are Accelerated by Ectopic BMI1 Expression
Source: Cancer Res Commun. 2026 Jan 20;6(1):152–64. doi: 10.1158/2767-9764.CRC-25-0580 (PMC12816948; doi:10.1158/2767-9764.CRC-25-0580)
Supplement: Supplementary Figure 11 — Key changes regulated by BMI1 in two OSCC models. [file crc-25-0580_supplementary_figure_11_suppsf11.docx]

**Supplementary Figure 11.** Key changes regulated by BMI1 in two OSCC models. Arrows depict the effect of BMI1 (either by ectopic overexpression or deletion) on these processes.
