# Supplementary material for: Key Early Changes in Oral Squamous Cell Carcinogenesis Are Accelerated by Ectopic BMI1 Expression
Source: Cancer Res Commun. 2026 Jan 20;6(1):152–64. doi: 10.1158/2767-9764.CRC-25-0580 (PMC12816948; doi:10.1158/2767-9764.CRC-25-0580)
Supplement: Supplementary Table 3 — Antibody list for Immunofluorescence/ Immunohistochemistry [file crc-25-0580_supplementary_table_3_suppst3.docx]

**Supplementary Table 3.** Antibody list for Immunofluorescence/ Immunohistochemistry

| **Target** | **Source** | **Company** | **Catalog #** | **Lot #** | **Dilution** | **RRID** |
| --- | --- | --- | --- | --- | --- | --- |
| BMI1 | Rabbit Monoclonal | Cell Signaling | 6964S | 3 | 1:100 | AB_10828713 |
| BMI1 | Mouse Monoclonal | Santa Cruz | sc-390443 | K1320 | 1:50  *1:50 (20 min) | AB_3674579 |
| 4-HNE | Rabbit Polyclonal | Abcam | ab46545 | GR3406160-2 | *1:1000  (3:30 min) | AB_722490 |
| GLUT1 | Rabbit Polyclonal | Abcam | ab14683 | 786962 | 1:200  *1:500 (5min) | AB_301408 |
| GPI1 | Rabbit Polyclonal | Protein  Tech | 15171-1-AP | 00007145 | *1:200 (3:30 min) | AB_2263537 |
| HIF1α | Mouse Monoclonal | Invitrogen | MA1-516 | YJ383208 | *1:100 (20 min) | AB_325431 |
| HIF1α | Mouse Monoclonal | BD Biosciences | 610959 | 1011250 | 1:50 | AB_398272 |
| Ki67 | Rabbit Monoclonal | Cell Signaling | 9129S | 3 | *1:400 (7 min) | AB_2687446 |
| PKM2 | Rabbit Polyclonal | Protein  Tech | 15822-1-AP | 00040553 | *1:200 (3:30 min) | AB_1851537 |
| SLC16A3 | Rabbit Polyclonal | Invitrogen | PA5-54413 | WB3204708 | *1:200 (3:30 min) | AB_2647363 |
| SOX9 | Rabbit Monoclonal | Cell Signaling | 82630S | 1 | *1:100 (7 min) | AB_2665492 |
| **SECONDARY** | **(IF)** |  |  |  |  |  |
| anti-mouse IgG, Alexa Fluor 594 | Goat Polyclonal | Invitrogen | A11005 | 2538976 | 1:500 | AB_2534073 |
| anti-rabbit IgG, Alexa FluorPlus 488 | Donkey Polyclonal | Invitrogen | A32790 | VC296619 | 1:500 | AB_2762833 |

*Dilution used for IHC and DAB incubation time
