# Supplementary material for: Key Early Changes in Oral Squamous Cell Carcinogenesis Are Accelerated by Ectopic BMI1 Expression
Source: Cancer Res Commun. 2026 Jan 20;6(1):152–64. doi: 10.1158/2767-9764.CRC-25-0580 (PMC12816948; doi:10.1158/2767-9764.CRC-25-0580)
Supplement: Supplementary Table 4 — List of primers used in qRT-PCR (Mouse Tissues) [file crc-25-0580_supplementary_table_4_suppst4.docx]

**Supplementary Table 4.** List of primers used in qRT-PCR (Mouse Tissues)

| **Primer** | **Sequence** | **Size (basepairs)** |
| --- | --- | --- |
| BMI1 (F) | 5'-GTCAGCTGATGCTGCCAATG-3' | 462 |
| BMI1 (R) | 5'-GCCTTGTCACTCCCAGAGTC-3' | 462 |
| 36B4 (F) | 5'-AGAACAACCCAGCTCTGGAGAAA-3' | 448 |
| 36B4 (R) | 5'-ACACCCTCCAGAAAGCGAGAGT-3' | 448 |
| HIF1A (F) | 5'-GGCAGCGATGACACAGAAAC-3' | 339 |
| HIF1A (R) | 5'-AGGCTGGGAAAAGTTAGGAGT-3' | 339 |
| SLC2A1 (F) | 5'-CAGCAGCTGTCGGGTATCAA-3' | 312 |
| SLC2A1 (R) | 5'-AGGACCAGGGCCTACTTCAA-3' | 312 |
