# Supplementary material for: Key Early Changes in Oral Squamous Cell Carcinogenesis Are Accelerated by Ectopic BMI1 Expression
Source: Cancer Res Commun. 2026 Jan 20;6(1):152–64. doi: 10.1158/2767-9764.CRC-25-0580 (PMC12816948; doi:10.1158/2767-9764.CRC-25-0580)
Supplement: Supplementary Table 5 — List of primers used in ChIP-qPCR (SCC-25 cells) [file crc-25-0580_supplementary_table_5_suppst5.docx]

**Supplementary Table 5.** List of primers used in ChIP-qPCR (SCC-25 cells)

| **Primer** | **Sequence** | **Size (basepairs)** |
| --- | --- | --- |
| HIF1A Promoter 1 (F) | 5'-CTTCTCTTCTCCGCGTGTGG-3' | 127 |
| HIF1A Promoter 1 (R) | 5'-TTTTCTTGTCGTTCGCGCC-3' | 127 |
| HIF1A Promoter 4 (F) | 5'-GGATCACCCTCTTCGTCGCT-3' | 127 |
| HIF1A Promoter 4 (R) | 5'-CCCCTCGTGAGACTAGAGAGA-3' | 127 |
| HPRT1 Control (F) | 5'-TCTCTGAGGAGATGCAGTCAG-3' | 177 |
| HPRT1 Control (R) | 5'-GGAGGGGCCTAGAAGTGGTA-3' | 177 |
| PTEN Control (F)* | 5'-GGAGGCAGCCGTTCGGAGGATTATT-3' | 201 |
| PTEN Control (R)* | 5'-GGAAATGGCTCTGGACTTGGCGGTA-3' | 201 |

*Primers for PTEN positive control from Song et al., 2009.
