# Supplementary material for: Key Early Changes in Oral Squamous Cell Carcinogenesis Are Accelerated by Ectopic BMI1 Expression
Source: Cancer Res Commun. 2026 Jan 20;6(1):152–64. doi: 10.1158/2767-9764.CRC-25-0580 (PMC12816948; doi:10.1158/2767-9764.CRC-25-0580)
Supplement: Supplementary Table 6 — guide RNA (gRNA) sequences used to knock out BMI1 in SCC-25 cells [file crc-25-0580_supplementary_table_6_suppst6.docx]

**Supplementary Table 6.** guide RNA (gRNA) sequences used to knock out BMI1 in SCC-25 cells

| **Name** | **Sequence** | **Exon Targeted** |
| --- | --- | --- |
| BMI1 A | 5'-AAUGGCUCUAAUGAAGAUAG-3' | Exon 6 |
| *BMI1 B | 5'-GAUUGAUGUCAUGUAUGAGG-3' | Exon 9 |
| BMI1 C | 5'-UAUAUCGUUAUAGCUGCCAA-3' | Intron 5 - Exon 6 |

*gRNA that resulted in the highest percentage of edited SCC-25 cells
