# Supplementary material for: Key Early Changes in Oral Squamous Cell Carcinogenesis Are Accelerated by Ectopic BMI1 Expression
Source: Cancer Res Commun. 2026 Jan 20;6(1):152–64. doi: 10.1158/2767-9764.CRC-25-0580 (PMC12816948; doi:10.1158/2767-9764.CRC-25-0580)
Supplement: Supplementary Table 7 — Primers used to amplify regions around expected edited sites (via PCR) for gRNAs employed in BMI1 knockouts (KO) [file crc-25-0580_supplementary_table_7_suppst7.docx]

**Supplementary Table 7.** Primers used to amplify regions around expected edited sites (via PCR) for gRNAs employed in BMI1 knockouts (KO)

| **Primer** | **Sequence** | **Exon Targeted** |
| --- | --- | --- |
| Forward for BMI1 A/C | 5'-GAGGGAAGACATTTTATATGGGGG-3' | Exon 6 |
| Reverse for BMI1 A/C | 5'-TCTTTGTTTACTTTCCGATCCAATC-3' | Exon 6 |
| *Forward for BMI1 B | 5'-TGAAGTTTCAGGAGTCTTTCTTTG-3' | Exon 9 |
| *Reverse for BMI1 B | 5'-CTCTGTATTTCAATGGAAGTGGACC -3' | Exon 9 |

*Sanger Sequencing revealed that BMI1B gRNA resulted in the highest percentage of edited cells
