# Supplementary material for: Key Early Changes in Oral Squamous Cell Carcinogenesis Are Accelerated by Ectopic BMI1 Expression
Source: Cancer Res Commun. 2026 Jan 20;6(1):152–64. doi: 10.1158/2767-9764.CRC-25-0580 (PMC12816948; doi:10.1158/2767-9764.CRC-25-0580)
Supplement: Supplementary Table 8 — Antibody list for Western Blotting [file crc-25-0580_supplementary_table_8_suppst8.docx]

**Supplementary Table 8.** Antibody list for Western Blotting

| **Target** | **Source** | **Company** | **Catalog #** | **Lot #** | **Dilution** | **RRID** |
| --- | --- | --- | --- | --- | --- | --- |
| BMI1 | Rabbit Monoclonal | Cell Signaling | 6964S | 3 | 1:1000 | AB_10828713 |
| GAPDH | Rabbit Polyclonal | ABclonal | AC027 | 3507443012 | 1: 1000 | AB_2769572 |
| GLUT1 | Rabbit Polyclonal | Abcam | ab14683 | 1080816-4 | 1: 4000 | AB_301408 |
| HIF1α | Mouse Monoclonal | BD Biosciences | 610959 | 1011250 | 1: 500 | AB_398272 |
